# *Staphylococcus* Great Britain and Ireland 2023 (StaphGBI 2023) Conference Report

**DOI:** 10.1099/acmi.0.000730.v3

**Published:** 2023-12-05

**Authors:** James P. O'Gara, Merve S. Zeden

**Affiliations:** ^1^​ Microbiology, School of Biological and Chemical Sciences, College of Science and Engineering, University of Galway, Galway, Ireland

**Keywords:** research community, StaphGBI 2023, *Staphylococcus*, UK and Ireland

## Abstract

Since 1997, *Staphylococcus* Great Britain and Ireland (StaphGBI) conferences have brought together the *Staphylococcus* research community in the UK and Ireland. The 12th StaphGBI conference, hosted by University of Galway 22–23 June 2023, was co-chaired by Dr Merve S. Zeden and Professor James P. O’Gara, supported by a local organizing committee of Chloe Hobbs-Tobin, Dr Rakesh Roy, Órla Burke and Aaron Nolan. Anchored by keynote speaker Professor Vinai Thomas, all other StaphGBI 2023 oral and post presentations were delivered by early career researchers. The conference attracted approximately 100 delegates, including 72 MRes/PhD students and postdoctoral fellows, 22 principal investigators and 4 exhibitors. The mix of scientists, clinicians and early career researchers stimulated excellent discussions on key issues and challenges in the *Staphylococcus* field. *Staphylococcus aureus* interactions with the host immune system, antimicrobial resistance (AMR) and new therapeutic approaches using antimicrobial peptides or metabolites, chronic wound and device-associated infections, and improving our understanding of staphylococcal genomics were common themes at StaphGBI 2023.

## Conference programme and oral presentations

The conference was divided into five sessions covering the breadth of staphylococcal research.

The opening session, Staphylococcal Genomics, was chaired by Dr Séana Duggan (University of Exeter). Michelle Rudden (Hull York Medical School) described the use of nanopore sequencing to taxonomically define the skin microbiome profile of two pig wound infection models as part of experiments to demonstrate that the novel bacteriophage-derived endolysin (XZ.700) selectively depletes *Staphylococcus aureus *, restoring microbiome diversity and promoting healing. Rebecca Man (University of Edinburgh) gave an interesting presentation on the importance of lateral transduction on *S. aureus* genome organization and diversity. J. J. Awodipe (University of Warwick) described research using dual RNA sequencing analysis to simultaneously analyse bacterial and host transcriptional responses during the initial stages of *S. aureus* osteoblast invasion and intracellular replication.

Session II, Staphylococcal Physiology and Metabolism, was chaired by Dr Rebecca Corrigan (University of Sheffield). Merve S. Zeden (University of Galway) described insights into the mechanistic basis for significantly increased methicillin-resistant *S. aureus* (MRSA) resistance to β-lactam antibiotics in a pentose phosphate pathway (*pgl*) mutant. Igor Kviatkovski (Imperial College London) reported on the identification of novel genetic factors that regulate c-di-AMP production in *S. aureus* using an elegant riboswitch-based biosensor. Stephen Garrett (Newcastle University) gave an excellent presentation on TslA, a novel ‘reverse’ toxin of the *S. aureus* type VII secretion system. Using beautiful microscopy images, Joshua Sutton (University of Sheffield) outlined the role of GpsB in *S. aureus* cell shape determination. Sean Brennan (University of Leicester) reported that the pleiotropic impact of copper on the *S. aureus* transcriptome, including global regulators, acts as a signal to alter virulence. The final presentation in this session by Imran Khan (University of York) summarized a comprehensive investigation of *S. aureus* peptide utilization, including the specific role of the proton-dependant oligopeptide transporter DtpT.

Session III, Staphylococcal Antimicrobial Resistance and New Therapeutics, was chaired by Dr Merve S. Zeden (University of Galway). First up, Arely Leyton (University College London) outlined the analysis of several putative aminotransferases predicted to be involved in the aspartate and the haem biosynthetic pathways, which may represent good drug targets. Edward Douglas (University of Bath) reported that structural enhancements of the 1,3,4 oxadiazoyl-based lipoteichoic acid synthase inhibitor 1771 significantly enhanced antimicrobial activity via a new phospholipid biosynthesis-inhibiting mechanism. Aaron Nolan (University of Galway) reported how analysis of purine salvage pathway mutants with increased MRSA resistance to β-lactam antibiotics helped to reveal the therapeutic potential of purine nucleosides as β-lactam adjuvants. Hawraa Shahrour (Royal College of Surgeons in Ireland) described the potent antibiofilm activity of hydrogels incorporating the novel antimicrobial agents ML8 (lipid derivative), d-Bac8C (antimicrobial peptide) and SH3Lys-ChapK (bacteriophage endolysin), with the potential to enhance wound infection therapeutics.

The keynote lecture for StaphGBI 2023 was delivered by Professor Vinai Thomas (University of Nebraska Medical Centre, Omaha). Professor Thomas is an expert on pathogen adaptations during stress and infection and his research aims to identify metabolic pathways or cellular targets of therapeutic importance that limit the ability of pathogen to adapt during infection. Current projects in his laboratory seek to understand how *S. aureus* and *Staphylococcus epidermidis* alter metabolism and energetics to optimize fitness during stress. In his lecture entitled ‘How *Staphylococcus aureus* Counters Host Organic Acid Defenses During Colonization’ Vinai memorably started with an illustration of two liquid flask cultures of *S. aureus* growing under different conditions, asking the question of how this pathogen can mitigate the toxicity of short-chain organic acids prevalent in its environment to successfully colonize human hosts. These acids, such as acetate, lactate and propionate, can be present in varying concentrations, depending on the colonization niche, sometimes reaching high millimolar levels. The lecture focused on the mechanisms underlying the toxicity of acetic acid, describing the cellular components corrupted by acetic acid, and delineating the strategies employed by *S. aureus* to sustain its fitness under acetic acid-induced stress. This beautifully and expertly delivered lecture was a tour de force of bacterial physiology, metabolism and genetics and reinforced the importance of discovery research in understanding the survival strategies of *S. aureus* against organic acids, which could pave the way for novel targeted therapeutics.

Session IV, Staphylococcal Infections and Pathogen–Host Interactions, was chaired by Professor Rachel McLoughlin (Trinity College Dublin). Leading out, Richard Allen (University of Warwick) used time lapse microscopy and other approaches to implicate the *S. aureus* type VII secretions system in control of host cell death pathways in macrophages. Toska Wonfor (University of Bath) summarized new insights into *S. aureus* complement evasion strategies derived from experiments using a modified serum bactericidal assay with a complement-susceptible *Escherichia coli* strain. Emily Stevens (University of Oxford) described research to investigate the facilitative impact of the microbiota of the nematode *Caenorhabditis elegans* on *S. aureus* infection. Simon Carlile (Trinity College Dublin) reported that *S. aureus* ‘trained’ mice generated through repeated exposure to the pathogen were better able to clear subsequent *E. coli* infections via an enhanced inflammatory response, suggesting a potential protective effect of *S. aureus* colonization. Lizzie Ledger (Imperial College London) described how adaptation of *S. aureus* to serum significantly reduces the ability of the immune system to detect and kill this pathogen through concealment of bound opsonins via peptidoglycan accumulation. In the final presentation of this session, Willow Fox (University of Edinburgh) described how, unlike human isolates, bovine isolates of *S. aureus* are adapted for growth in milk and the identification of key genes and pathways required for this host adaptation.

Session V, Staphylococcal Virulence Mechanisms and Biofilms, was chaired by Professor Eoghan O'Neill (Royal College of Surgeons in Ireland). Dora Bonini (University of Bristol) opened this session with a wonderful talk on the role of the small membrane protein MspA in regulating lipoteichoic acid biosynthesis by interfering with the interactions between the LtaA and LtaS enzymes. Ciara Furlong (Dublin City University) described how understanding interspecies interactions between *S. aureus* and non-*albicans Candida* species can be exploited to identify novel anti-biofilm treatments. Mary Turley (Trinity College Dublin) reported that bile, which is often aspirated into the lungs of patients with cystic fibrosis, can activate the *icaADBC* operon and promote polysaccharide-type biofilm production in *S. aureus *, with implications for neutrophil clearance and antibiotic tolerance. Having chaired the first session, Dr Seána Duggan (University of Exeter) bookended the conference with an excellent presentation on the role of copper availability in shaping *Staphylococcus–Candida* interactions in multispecies biofilms.

## Prize winners

There was widespread agreement that the quality of the data presented by all of the speakers and delivery of the presentations were exemplary. Following difficult deliberations, the judges charged with selecting the best oral presentations by a postgraduate student and a postdoctoral fellow, Professor Tim Foster (Trinity College Dublin) and Dr Simon Clark (University of Reading) selected Aaron Nolan (PhD student, University of Galway) and Joshua Sutton (Postdoctoral Fellow, University of Sheffield). The organizing committee’s prize for the best oral presentation went to Dora Bonini (PhD student, University of Bristol).

A highlight of the conference was the poster sessions. The quality of research on display was outstanding and was a wonderful opportunity to catch up with old friends, make new friends and hear about the exciting work ongoing in *Staphylococcus* research labs throughout Ireland and the UK. The judges, Professor Ruth Massey (University College Cork), Dr Andrew Edwards (Imperial College London) and Professor Julie Morrissey (University of Leicester), selected Sean Cahill (PhD student, Trinity College Dublin) and Lucy Urwin (Postdoctoral Fellow, University of Sheffield) for poster presentation prizes.

## Concluding remarks, general themes that emerged during the conference, and StaphGBI 2025

The mix of scientists, clinicians and early career researchers stimulated excellent discussions on key issues and challenges in the *Staphylococcus* field. *S. aureus* interactions with the host immune system, AMR and new therapeutic approaches using antimicrobial peptides or metabolites, chronic wound and device-associated infections, and improving our understanding of staphylococcal genomics were common themes at StaphGBI 2023.

University of Galway is a United Nations Sustainable Development Goal (SDG) champion and the conference was plastic-free, with a water filling station and minimal printing of conference materials. The conference programme was available online via a QR link from the name badges.

Beautiful late June weather allowed attendees to enjoy vibrant Galway city, as well as Connemara to the west and the Burren to the south.

Finally, following expressions of interest from several universities, conference delegates were delighted by the announcement that Professor Julie Morrissey and a local organizing committee will host StaphGBI at the University of Leicester in 2025.

